# Tobacco and Menthol flavored electronic cigarettes induced inflammation and dysregulated repair in lung fibroblast and epithelium

**DOI:** 10.21203/rs.3.rs-3037297/v1

**Published:** 2023-06-12

**Authors:** Qixin Wang, Joseph H Lucas, Cortney Pang, Ruogang Zhao, Irfan Rahman

**Affiliations:** Department of Environmental Medicine, University of Rochester Medical Center, Rochester, NY, USA.; Department of Environmental Medicine, University of Rochester Medical Center, Rochester, NY, USA.; Department of Environmental Medicine, University of Rochester Medical Center, Rochester, NY, USA.; Department of Biomedical Engineering, University at Buffalo, Buffalo NY, USA.; Department of Environmental Medicine, University of Rochester Medical Center, Rochester, NY, USA.

**Keywords:** Menthol, nicotine-free, tobacco, repair, injury, inflammation, ENDS

## Abstract

**Background:**

Electronic cigarette (e-cig) vaping has increased in the past decade in the US, and e-cig use is misleadingly marketed as a safe cessation for quitting smoking. The main constituents in e-liquid are humectants, such as propylene glycol (PG) and vegetable glycerine (VG), but different flavoring chemicals are also used. However, the toxicology profile of flavored e-cigs in the pulmonary tract is lacking. We hypothesized that menthol and tobacco-flavored e-cig (nicotine-free) exposure results in inflammatory responses and dysregulated repair in lung fibroblast and epithelium.

**Method:**

We exposed lung fibroblast (HFL-1) and epithelium (BEAS-2B) to Air, PG/VG, menthol flavored, or tobacco-flavored e-cig, and determined the cytotoxicity, inflammation, and wound healing ability of the cells in a microtissue chip model.

**Results:**

After exposure, HFL-1 showed decreased cell number with increased IL-8 levels in the tobacco flavor group compared to air. BEAS-2B also showed increased IL-8 secretion after PG/VG and tobacco flavor exposure, while menthol flavor exposure showed no change. Both menthol and tobacco-flavored e-cig exposure showed decreased protein abundance of type 1 collagen (COL1A1), α-smooth-muscle actin (αSMA), and fibronectin as well as decreased gene expression level of αSMA (*Acta2*) in HFL-1. After tobacco flavor e-cig exposure, HFL-1 mediated wound healing and tissue contractility were inhibited. Furthermore, BEAS-2B exposed to menthol flavor showed significantly decreased gene expression of *CDH1, OCLN,* and *TJP1*.

**Conclusion:**

Overall, tobacco-flavored e-cig exposure induces inflammation in both epithelium and fibroblasts, and tobacco-flavored e-cig inhibits wound healing ability in fibroblast.

## Background

Electronic nicotine delivery systems (ENDS), also known as electronic cigarettes (e-cigs) are devices that generate aerosols to mimic cigarette smoking, and e-cigs are marketed broadly as a smoking cessation tool [1–4]. The e-liquid mixture usually contains humectants, nicotine, and other additives. The humectant used in e-liquid is a mixture of propylene glycol (PG) and/or vegetable glycerin (VG), which are substances generally recognized as safe (GRAS). Other additives added to e-liquids include different flavoring chemicals, which were initially added to improve the taste and ease the throat hit, butunexpectedly attracted younger generations [5]. In 2022, around 2.5 million high-school and middle-school students are active e-cig users, and 85% of products are flavored e-cigs [6]. Although FDA issued a series of policies and rules to regulate different flavored e-cigs in 2020, tobacco and menthol flavored e-cigs are still legally available on the market, which showed an unexpected increase in consumption after the flavor ban [7]. Since nicotine is an addictive substance in e-liquid, various vendors have provided nicotine-free e-cigs with both menthol and tobacco-flavored e-cigs and advertised them as non-addictive products for smoking cessation. However, our and others’ previous reports have shown that e-cig aerosol without nicotine showed lung inflammatory responses, impaired lung function, and dysregulated tissue repair [8–11]. We also found that 1-month e-cig exposure showed dysregulated expressions of matrix metalloproteinases (MMPs), A disintegrin and metalloprotease (ADAMs), and collagens which are responsible for extracellular matrix (ECM) remodeling, wound healing and repair [8, 9, 11]. Although studies showed that e-cig exposure induced inflammatory responses and potential dysregulated repair, no study currently focuses on how menthol and tobacco flavored e-cigs cause dysregulated wound healing.

Wound healing in the lung is usually initiated after injury on the lung epithelium, and lung fibroblasts migrate to the injury site and accelerate epithelial repair [12]. Considering e-cig vaping is a constant habit, other environmental hazards, such as viral infection, might occur during e-cig vaping. Dysregulated wound healing due to e-cig exposure could be one of the reasons for lung injury exacerbation, as observed in our prior report showing that e-cig vaping augments IAV infection-induced lung injury [13–15]. Lung fibroblasts play a critical role in tissue wound healing and repair, differentiating from fibroblast to myofibroblast during the repair process, which will be accompanied by overexpression of ECM components, such as collagen, fibronectin and α-smooth muscle actin (αSMA). The overexpressed ECM components increase tissue contractility and generate new ECM that could support the epithelium and accelerate the epithelium repair [12, 16]. Our previous studies showed that flavored e-cig exposure or nicotine treatment inhibited TGF-β induced fibroblast differentiation and induced premature senescence and inflammatory response in a dose-dependent manner [17, 18]. No study focused on how menthol and tobacco-flavored e-cigs (nicotine free) affect the wound healing process mediated by lung fibroblast, which is especially important since menthol and tobacco-flavored e-cig are currently the only legally allowed e-cigs on the market.

In this study, we hypothesized that menthol and tobacco-flavored e-cig (nicotine-free) exposure induces inflammatory responses and inhibits wound healing ability. We exposed human lung fibroblast (HFL-1) and human bronchial epithelial cell (BEAS-2B) to PG/VG, PG/VG + Menthol, and PG/VG + Tobacco to determine the impact of how flavored e-cig exposure disrupts wound healing mediated by lung fibroblast and the inflammatory response from both lung fibroblast and epithelium. We also applied a microtissue chip that can reflect the altered contractility of fibroblast during wound healing response following e-cig exposure.

## Methods

### Cell culture

Human fetal lung fibroblast (HFL-1, Cat#: CCL-153) and human bronchial epithelial cell (BEAS-2B, Cat#: CRL-9609) were purchased from the American Type Culture Collection (ATCC) and maintained in DMEM/F12K medium with 10% FBS (Cat#: 10082147; Thermo Fisher Scientific) for HFL-1, and 5% FBS for BEAS-2B, and total 1% Penicillin-Streptomycin-Glutamine (Cat#: 103–78016; Thermo Fisher Scientific) with 5% CO_2_ and 95% humidity. HFL-1 and BEAS-2B were seeded at 30,000 cells/cm^2^ in 6 well plate for 1 day, and HFL-1 were starved in FBS-free medium while BEAS-2B were in 1% FBS medium overnight. Then, the cells were exposed to air, PG/VG (50:50), PG/VG + menthol flavor (nicotine free), and PG/VG + tobacco flavor (nicotine free) the next day. After exposure, cells were either lysed for protein and RNA isolation or fixed with ice-cold methanol for immunofluorescence staining.

### E-cig device, e-liquids, and e-cig exposure

The e-cig device used is the Joytech eVIC VTCmini with a 0.15Ω atomizer/coil (Kanger Tech). The e-liquids containing PG/VG (50:50), PG/VG + menthol flavor (nicotine free), and PG/VG + tobacco flavor (nicotine free) were procured from a local vendor. The air pump, connecting tubing, and atomizers were changed in between groups. E-cig aerosol was generated and pumped into an Enzyscreen chamber at a rate of 2 puffs per minute for 2 minutes, and another 8 mins were allowed for the e-cig aerosol to deposit. The puffing profile was based on the realistic topography with 3.3s/puff, 26.7s interval, and 70mL puff volume.

### ELISA

After 2 days of exposure, the conditioned medium was collected and stored at −80°C. The levels of IL-6 (Catalog# CHC1263, Thermo Fisher Scientific) and IL-8 (Catalog# CHC1303, Thermo Fisher Scientific) were detected in the conditioned medium from both HFL-1 and BEAS-2B, while TGF-β (Catalog# DY240, R&D System) was detected only in HFL-1, according to the manufacturer’s manuals.

### RNA isolation and qRT-PCR

After 2 days of exposure, cells were washed with PBS twice, and lysed in 700 μL QIAzol reagent (Cat#:79306, Qiagen) for 15 mins at room temperature, then collected into a 1.7mL tube. Then, 150 μL chloroform was added to the sample, and vortexed for 10s. The mixtures were centrifuged at 20,000g for 15 mins at 4°C. The aqueous phase was transferred to a new tube, and 200 μL of isopropanol was added to the samples and mixed gently. The mixtures were incubated at −20°C for 3 hrs, and then spun down at 20,000g for 15 mins at 4°C. The isopropanol, was removed, the RNA pellets were washed with 75% EtOH, and then centrifuged at 20,000g for 15 mins at 4°C. The EtOH was removed, and the RNA pellet was resuspended with Rnase-free water. RNA concentration and quality were checked by Nano-drop spectrophotometer (ND-1000, NanoDrop Technologies). A total of 200 ng of RNA was used for reverse transcription via RT2 First Strand Kit (Cat# 330401, Qiagen). Synthesized cDNA was diluted 6 times, and used for real-time PCR quantification by using SYBR green expression master-mix (Cat# 330509, Qiagen) in BioRad CFX96 qPCR machine. All the primers were purchased from BioRad: COL1A1 (Human, qHsaCEP0050510), ACTA2 (Human, qHsaCIP0028813), FN1 (Human, qHsaCEP0050873), CDH1 (Human, qHsaCID0015365), CDH2 (Human, qHsaCID0015189), VIM (Human, qHsaCED0042034), TJP1 (Human, qHsaCID0018062), OCLN (Human, qHsaCED0038290), SERPINE1 (Human, qHsaCID0006432), and GAPDH (Human, qHsaCEP0041396). The thermal cycle for qRT-PCR was 10 min at 95°C, then 95°C, 15 s, and 60°C, 1 min for 40 cycles, with fluorescence intensity measurement at the end of 1 min incubation at 60°C. The melting curve was performed when the 40 cycles were finished. The raw Cq value will be used, and the relative change fold will be calculated via 2^−ΔΔCt^ methods with GAPDH as the housekeeping gene.

### Protein isolation and Western blot

Cells were lysed in RIPA buffer and isolated protein was quantified by Pierce BCA Assay Kit (Cat#: 23227, Thermo Fisher Scientific). Total 20 μg of protein was loaded in each lane, and run through 10% sodium dodecyl sulfate–polyacrylamide gel electrophoresis (SDS-PAGE). The protein was then transferred onto a nitrocellulose membrane (Cat# 1620112, BioRad). The membrane was washed with Tris-buffered saline containing 0.1% Tween 20 (TBS-T) for 10 mins, and then blocked with 5% non-fat milk for 1hr at room temperature. Primary antibodies: anti-Fibronectin (1:1000, ab, Abcam), Anti-α-Smooth Muscle Actin (α-SMA) (1:1000, A2547, Sigma), anti-COL1A1 (1:1000, NBP1–30054, Novus Biologicals), anti-vimentin (1:1000, ab92547, Abcam), anti-E-Cadherin (1:1000, 3195, Cell Signaling), anti-N-Cadherin (1:1000, ab76011, Abcam), Anti-Occludin (1:1000, ab216327, Abcam), anti-ZO1 (1:1000, ab221547, abcam), anti-PAI1 (1:1000, ab222754, abcam), and GAPDH (1:1000, ab9482, Abcam), were incubated overnight at 4°C. The following day, the primary antibody was removed, and the membrane was washed 4 times, 15 mins each, with TBS-T. Next, the secondary antibody (goat-anti-rabbit, 1:5000, #1706515, BioRad; Rabbit Anti-Mouse, 1:5000, ab6728, Abcam) was incubated for 1 hr at room temperature. After, the membrane was washed with TBS-T for 4 times, 15 mins each, and then detection of the signal with Pierce ECL Western Blotting Substrate (Cat#: 32106, Thermo Scientific) via Bio-Rad ChemiDoc MP imaging system was done. ImageLab software (BioRad) was used to normalize the densitometry and calculate the change fold based on the air group. GAPDH was used as the endogenous control to normalize for sample variation.

### Immunofluorescence staining

Cells after e-cig exposure for 2 days were fixed with pre-chilled methanol for 10 mins at 4°C, and then wash with TBS for 5 mins, 3 times. Then, the cells were blocked with 10% normal horse serum for 1 hr at room temperature and incubated with anti-COL1A1 (1:100, NBP1–30054, Novus Biologicals) for 16 hr at 4°C. The primary antibody was removed and then washed with TBS for 4 times, 5 mins each, and then incubated with goat anti-rabbit IgG (H + L) secondary antibody Alexa Fluor 488 (1:1000, Catalog # A-11008, ThermoFisher) for 1 hr at room temperature. Cells were washed with TBS for 4 times, 5 mins each, and then stained with Hoechst 33342 (Cat# H3570, Thermo Fisher Scientist) for 10 mins. Cells were kept in TBS, and stored at 4°C in the dark to avoid the light under fluorescence imaging. EVOS fluorescence microscopy was used to visualize the nuclear and stained COL1A1.

### Wound healing assay

Confluent HFl-1 cells were “scratched” by dragging a 200 μl micropipette tip in a single motion across the center of the well. Only the wells, where cells had well-defined edges were included in the study. Immediately following the scratch, images was taken by Cytation 5 system and then exposed to air, PG/VG, menthol or tobacco-flavored e-cigs. The same scratched area was imaged 24, 48, and 72 h post- exposure. Wound or scratch area was calculated in ImageJ.

### Microtissue seeding and contraction force measurement

The microtissue device was a polydimethylsiloxane (PDMS) based micropillar arrays in a P35 petridish. The fabrication of microtissue devices and microtissue seeding were described in our previous study [19]. Briefly, the microtissue device was sterilized with 70% EtOH for 15 mins, under UV overnight, then treated with Pluronic F-127 (P2443, Sigma) for 10 mins to avoid cell adhesion to the PDMS surface. HFL-1 was mixed with 3 mg/mL collagen type-I (rat tail, Corning) and 10% v/v Matrigel (356231, CORNING), and BEAS-2B were mixed with 2 mg/mL collagen. The mixtures with cells were then centrifuged with the device together at 1200 RPM for 2 mins at 4°C. The excess mixtures were carefully removed and then polymerized at 37°C. The device was maintained in the respective culture medium in an incubator with 5% CO^2^ and 95% humidity. The microtissue is fully formed in 2 days of culture, and the bottom and top of the micropillar images were taken before the e-cig exposure and for 2 days post-exposure. The contraction force was determined by the deflection position of the micropillar as described. The contraction force F = kδ, where δ is the deflection distance of both pillars: δ = (δ_1_ + δ_2_)/2, and k = 0.9 μN/μm, which is the spring constant materials. All the pictures of microtissues were taken with an Olympus CKX41 microscope.

### Statistical analysis

All the data were visualized through GraphPad Prism software (V.9.0), and significance was calculated via one-way ANOVA or student’s T-test. All the data were presented as mean ± SEM, and p < 0.05 was considered statistically significant.

## Results

### Tobacco-flavored e-cig exposure induced inflammation in HFL-1

**Figure 1:**
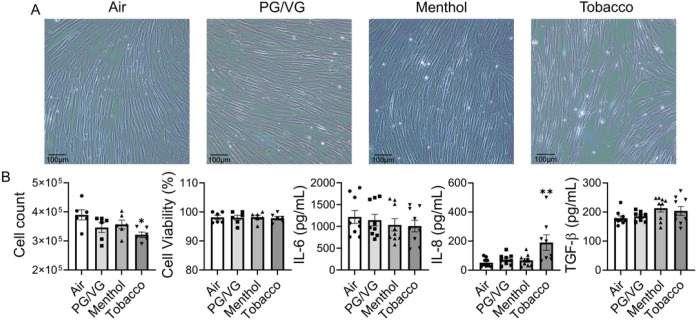
Tobacco flavored e-cig induced inflammatory responses in lung fibroblast. HFL-1 cells exposed to air, PG/VG, or tobacco flavored e-cig for 10 mins, and then cultured for 2 days. (A). Representative pictures of HFL-1 cells were taken under 20x microscope. (B). Cell number and viability was measured by AO/PI staining, and conditioned medium was collected for IL-6, IL-8 and TGF-β analysis. Data presented as mean± SEM (n = 6–9. * P < 0.05, ** P < 0.01, vs air).

To understand the cytotoxicity of tobacco and menthol-flavored e-cig, HFL-1 were exposed to PG/VG, menthol-flavored, and tobacco-flavored e-cig for 2 days. Significantly decreased cell number was noticed following tobacco-flavored e-cig exposure compared to air, and there is some gap in between the cells (Fig. 1). Although there is a decreased trend of cell number after PG/VG and menthol flavored e-cig exposure, no significant difference compared to the air group (Fig. 1B). Among all groups, there is no significant difference in cell viability (Fig. 1B). When we are detecting the levels of IL-6 and IL-8 released in the condition medium, significantly increased IL-8 was noticed after tobacco flavor e-cig exposure, while PG/VG and menthol flavor exposure showed no difference compared to air (Fig. 1B). No significant difference was detected in the level of IL-6 in the conditioned medium among the different groups (Fig. 1B). We also tested the levels of TGF-β released in condition medium among the different groups, no significant difference was detected (Fig. 1B).

### Menthol and tobacco-flavored e-cig exposure inhibit fibroblast differentiation markers

**Figure 2:**
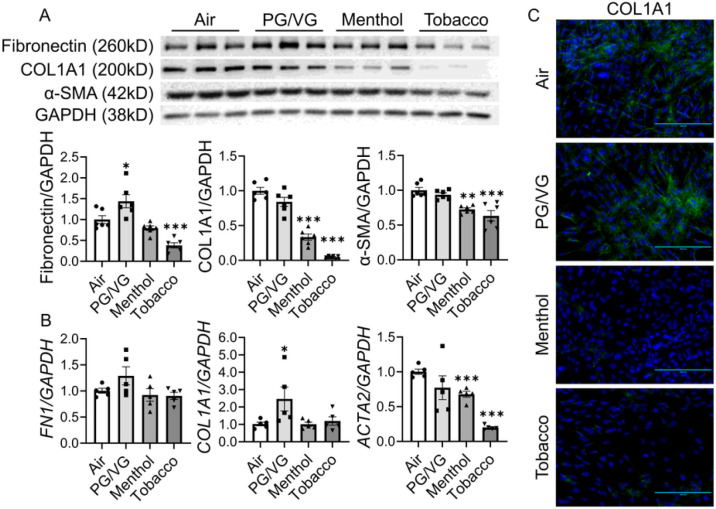
Tobacco flavored e-cig inhibited fibroblast differentiation markers. HFL-1 cells exposed to air, PG/VG, and tobacco flavored e-cig for 10 mins, and then cultured for 2 days. (A) cells were lysed and protein was isolated fro western blotting, fibronectin, COL1A1, and αSMA were analyzed, GAPDH was used as the endogenous control. (B). Cells were fixed, and stained with COL1A1, the fluorescence intensity was measured by EVOMS. (C). RNA was isolated from cells, and *FN1, COL1A1,* and *ACTA2* were measured by qRT-PCR, GAPDH was used as the endogenous control. Data presented as mean± SEM. (n = 5–6. * P < 0.05, ** P < 0.01, *** P < 0.001 vs air). Scale bar = 200 μm

We also isolated RNA and protein from HFL-1 after e-cig exposure and measured the expression levels of fibronectin, type 1 collagen, and α-SMA (Fig. 2). After exposure, the protein abundance of fibronectin was significantly up-regulated in the PG/VG group, while significantly decreased in the tobacco flavor group, compared to the air group (Fig. 2A and Suppl Fig. 1 with full blots). Significantly decreased protein expression levels of COL1A1 and α-SMA after menthol and tobacco flavored e-cig exposure were observed (Fig. 2A). Similarly, we also noticed a significantly decreased transcript level of *ACTA2* after menthol and tobacco-flavored e-cig exposure (Fig. 2B). Significantly increased gene expression of *COL1A1* was increased after PG/VG group compared to the air group, while no significant differences were found between menthol vs air, or tobacco vs air (Fig. 2B). Non-significant increased trend was noticed in the RNA level of *FN1* in PG/VG group compared to air group, and there is no altered gene expression in the menthol or tobacco exposure group compared to air group (Fig. 2B). Protein abundance and distribution of COL1A1 was also detected by immunofluorescence staining, which showed uniform distribution of COL1A1 in either air group or PG/VG exposed group, while diminished protein expression of COL1A1 was noticed after menthol and tobacco flavored e-cig exposure (Fig. 2C).

### Tobacco-flavored e-cig exposure inhibit wound healing ability and contractility in HFL-1

**Figure 3:**
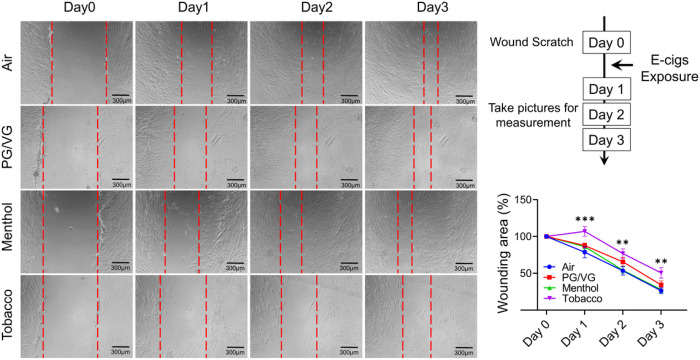
Tobacco flavored e-cigs inhibited wound healing mediated by lung fibroblast. HFL-1 cells were scratched and then exposed to air, PG/VG, or tobacco flavored e-cig for 10 mins, and then cultured for 2 days. The scratched wounds were monitored by taking pictures under the microscope daily. The same position has been selected by recording the coordinates from Cytation 5 imaging system. Data presented as mean± SEM. (n = 11–12. * P < 0.05, ** P < 0.01, vs air).

**Figure 4:**
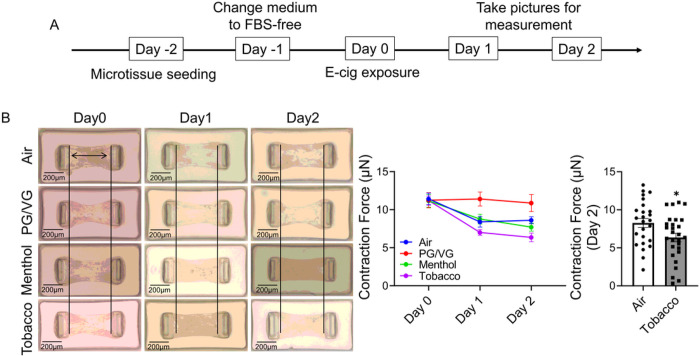
Tobacco flavored e-cig exposure decreased the contraction force of HFL-1 formed tissue. (A). HFL-1 cells were used to form microtissue first, then exposed to air, PG/VG, and tobacco flavored e-cig for 10 mins, and followed with 2 days culture. (B). The individual pictures of microtissue were taken by microscope for measurement of contraction force. Data presented as mean± SEM. (n ≥ 10. * P < 0.05 vs Air).

Since the fibroblast differentiation markers were inhibited after menthol and tobacco-flavored e-cig exposure, we would like to determine the wound healing ability of HFL-1 after menthol and tobacco-flavored e-cig exposure. We generated the wound scratch before e-cig exposure, and then exposed the scratched cells to PG/VG, menthol-flavored e-cig, and tobacco-flavored e-cig. It is evident that tobacco-flavored e-cig exposure slowed down the healing rate of the migration of fibroblasts into the wounded area, while no difference after PG/VG and menthol-flavored e-cig exposure was seen (Fig. 3). We also applied HFL-1 to form a microtissue for measuring the differentiated contractility after exposure to PG/VG, menthol-flavored, and tobacco-flavored e-cigs (Fig. 4A). Decreased contraction force was observed in air, menthol, and tobacco-flavored e-cig exposure groups after exposure, while the PG/VG group showed no altered contraction force during 2 days of culture (Fig. 2B). At day 2 post-exposure, tobacco-flavored e-cig exposure showed a significantly decreased contraction force compared to the air group (Fig. 2B).

### Flavored e-cig exposure induced inflammatory responses and epithelial-mesenchymal transition (EMT) in BEAS-2B

**Figure 5:**
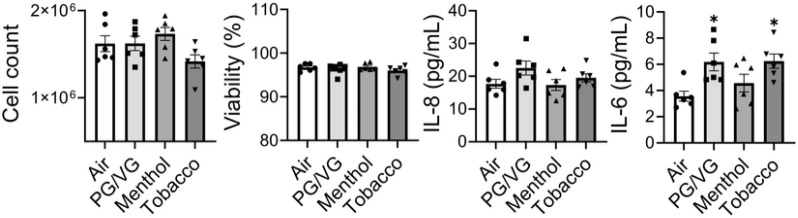
Unflavored and Tobacco flavored e-cig induced inflammatory responses in lung bronchial epithelium. BEAS-2B cells exposed to air, PG/VG, and tobacco flavored e-cig for 10 mins, and then cultured for 2 days. Cell number and viability was measured by AO/PI staining, and conditioned medium was collected for IL-6 and IL-8 analysis. Data presented as mean± SEM (n = 6. * P < 0.05 vs air).

**Figure 6:**
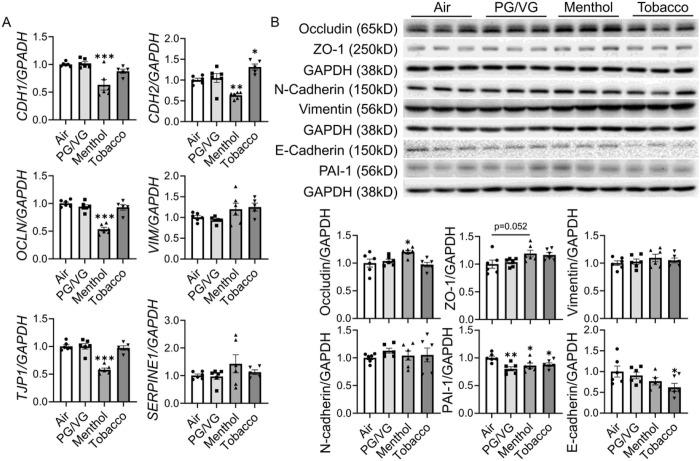
Flavored e-cig dysregulated EMT in lung bronchial epithelium. BEAS-2B cells exposed to air, PG/VG, and tobacco flavored e-cig for 10 mins, and then cultured for 2 days. (A) cells were lysed and RNA was isolated. The gene expression levels of *CDH1, CDH2, OCLN, VIM, TJP1,* and *SERPINE1* were measured by qRT-PCR, and *GAPDH* was used as the endogenous control. (B), Protein was isolated and expression levels of Occludin, ZO-1, Vimentin, N-cadherin, PAI-1, and E-cadherin were measured by western blot. GAPDH was used as the endogenous control for both RNA and protein normalization. Data presented as mean± SEM. (n = 5–6. * P < 0.05, ** P < 0.01, *** P < 0.001 vs air).

We also exposed BEAS-2B to PG/VG, menthol flavored, and tobacco flavored e-cig for 2 days, no significant difference was observed in cell count, cell viability, and IL-8 level among different condition groups (Fig. 5). The level of released IL-6 was upregulated after PG/VG and tobacco-flavored e-cig exposure compared to the air group (Fig. 5). RNA and protein were also isolated to identify EMT activation after e-cig exposure (Fig. 6). The gene expression of *CDH1, OCLN1, TJP1* and *CDH2* were decreased after menthol-flavored e-cig exposure compared to the air group, and non-significant increased transcript levels of *VIM* and *SERPINE1* were identified after the menthol group compared to the air group (Fig. 6A). Tobacco-flavored e-cig exposure showed increased gene expression of *CDH2* while no alteration in other gene expressions (Fig. 6A). There was no difference in protein abundance of vimentin, n-cadherin, and ZO-1 in PG/VG, menthol and tobacco groups compared to air group, while a slightly increased protein level of ZO-1 was identified after menthol-flavored e-cig exposure without significance (Fig. 6B). Increased protein abundance of occludin was found after menthol-flavored exposure, while decreased protein expressions of PAI-1 and E-cadherin were identified after tobacco-flavored e-cig exposure (Fig. 6B, and Suppl Fig. 1 with full blots). Moreover, decreased protein abundance of PAI-1 was also noticed after PG/VG and menthol-flavored e-cig exposure (Fig. 6B).

## Discussion

E-cig vaping has been proven to induce lung inflammation and potential tissue remodeling either in acute or chronic exposed mice, and adverse health effects occurred regardless of whether nicotine existed in e- liquid [8, 9]. Various merchandise have been labeled as nicotine-free e-cigs with various flavors to minimize the harmful effects of nicotine vaping, which have been established by various models [20]. To investigate the health risk of nicotine-free products, our recent study showed that both menthol and tobacco flavored e-cig, either with or without nicotine, presented with suppression on immune and inflammatory responses in mice [21]. Another study showed that nicotine free e-cig aerosol exposure induced inflammation responses in small airway epithelium [22]. Interestingly, exposure to nicotine-free e-cigs showed different results *in vivo* and *in vitro*, indicating that immune responses to e-cig aerosol are cell type-specific. In this study, we identified that lung fibroblast and epithelium exposed to nicotine free tobacco flavored e-cig showed increased inflammatory responses and both tobacco and menthol flavored e-cig exposure inhibited wound healing ability.

Our results showed that tobacco-flavored e-cig exposure decreased cell number and upregulated released IL-8 levels. Inflammatory responses were induced by tobacco-flavored e-cig while PG/VG exposure showed no significant difference compared to the air control. Our results indicate that flavoring chemicals used to prepare tobacco flavored e-cigs induce inflammation and cytotoxicity to lung fibroblast, while the humectant does not caue a response. Our recent study showed that one of the tobacco flavoring chemicals, eugenol, showed inflammatory activation on macrophages in a dose-dependent manner [23]. Other tobacco flavoring chemicals, such as coumarin, pentanedione, and maltol, have also been tested and showed significant cytotoxicity and induced oxidative stress in monocytes [24–26]. Previous studies further corroborate our results showing that tobacco flavoring chemicals used in tobacco-flavored e-cig dominated the inflammatory responses, while the humectant, PG/VG, showed no effect in activating inflammatory progression. However, we also noticed that both PG/VG and tobacco-flavored e-cig exposure showed increased IL-8 levels from lung epithelium. The inflammatory response from lung epithelium was from PG/VG itself, while tobacco flavoring chemicals used in e-liquid were not contributing to inflammation.

Other than inflammation, our previous study also showed that flavored e-cig exposure induced dysregulated repair and premature senescence in lung fibroblast [17]. In this study, we tested the wound healing ability of lung fibroblast after being exposed to menthol and tobacco-flavored e-cig. We noticed that the exposure of PG/VG showed increased gene and protein levels of fibronectin and COL1A1, which are the hallmarks of fibroblast differentiation. Our results agreed with a previous study that treatment of PG/VG increased the level of secreted COL1A1, which is even comparable to the TGF-β treatment group [18]. We also showed PG only exposure either acutely or chronically, showed potential dysregulated repair and remodeling in mice lung [8, 9]. Another report described that e-cig users showed logical trend of development of small airway fibrosis [22]. Both human and mouse models have indicated that e-cig exposure could lead to the development of fibrotic diseases while our study pointed that humectants used in e-liquid might be one of the major reasons.

Although the PG/VG exposure showed the activation of fibroblast differentiation which is required during wound healing, menthol and tobacco-flavored e-cig exposure showed significant inhibition of the protein levels of fibronectin and COL1A1, and both protein and gene abundance of α-SMA. Clearly, flavoring chemicals used in both menthol and tobacco-flavored e-liquid inhibit the differentiation markers, indicating inhibited wound healing ability. The scratch assay and microtissue chips applied in this study further confirmed that tobacco-flavored e-cig inhibited the wound healing ability mediated by lung fibroblast. Other studies also described that e-cig exposure slows the wound healing process. We have shown that nicotine treatment could inhibit the TGFβ-induced fibroblast differentiation and wound recovery [18]. Another report described that mint, menthol, vanilla, and fruit-flavored e-cig inhibited wound healing mediated by endothelium [27]. Exposure to e-cig aerosol also slowed the wound healing process on dermal cells, which showed even worse wound recovery than exposure to conventional cigarette smoke [28]. There are limited studies demonstrating the impact of e-cig vaping on dysregulated repair and wound healing, especially nicotine free, flavored e-cigs. This study indicated that tobacco flavored nicotine free e-cig exposure inhibits the wound healing process, fibroblast differentiation, and decreased fibroblast contractility. The inhibited wound healing process due to e-cig vaping could exacerbate lung injury that occurs as a second hit.

Previous research have shown that prior e-cig vaping decreased the survival rate of mice after IAV infection with more bodyweight loss compared to air group [13]. It is clear that IAV infection induced inflammation and immune responses were augmented after e-cig exposure [13]. Similar trends were also identified from the human *ex vivo* model that precision-cut lung slices (PCLS) from healthy donors showed fewer inflammation responses after IAV infection than PCLS from e-cig users [29]. As expected, prior e-cig vaping increased the severity of SARS-CoV-2 infection [30]. It is well known that e-cig exposure results in inflammatory responses, which is one of the reasons for lung injury exacerbation after IAV infection. Our study showed that inhibited wound healing ability after e-cig could delay the injury repairing and serve as one of the factors for causing prolonged lung damage and contribute to the development of chronic lung diseases.

## Conclusion

In conclusion, our results showed that tobacco-flavored nicotine-free e-cig exposure induced inflammation and cytotoxicity in lung fibroblast and epithelium and inhibited wound healing ability with decreased fibroblast differentiation markers and contractility. Inhibited wound healing capacity and pro-inflammatory responses induced by flavored e-cig exposure could be a key factor of lung injury exacerbation when challenged by other environmental hazards.

## Abbreviations

e-cig: Electronic cigarette
PG: Propylene glycol
VG: Vegetable glycerine
COL1A1: Type 1 collagen
αSMA: α-smooth-muscle actin
ENDS: Electronic nicotine delivery systems
GRAS: Generally recognized as safe
MMPs: Matrix metalloproteinases
ADAMs: A disintegrin and metalloprotease
ECM: Extracellular matrix
HFL-1: Human lung fibroblast
BEAS-2B: Human bronchial epithelial cell
PCLS: Precision-cut lung slices
